# Integration of statistical shape modeling and alternating interpolation-based model tracking technique for measuring knee kinematics* in vivo* using clinical interleaved bi-plane fluoroscopy

**DOI:** 10.7717/peerj.15371

**Published:** 2023-06-14

**Authors:** Hsuan-Yu Lu, Cheng-Chung Lin, Kao-Shang Shih, Tung-Wu Lu, Mei-Ying Kuo, Song-Ying Li, Horng-Chaung Hsu

**Affiliations:** 1 Department of Biomedical Engineering, National Taiwan University, Taipei, Taiwan, R.O.C.; 2 Department of Electrical Engineering, Fu-Jen Catholic University, New Taipei, Taiwan, R.O.C.; 3 Department of Orthopedics, Shin Kong Wu Ho-Su Memorial Hospital, Taipei, Taiwan, R.O.C.; 4 Department of Orthopaedic Surgery, School of Medicine, National Taiwan University, Taipei, Taiwan, R.O.C.; 5 Department of Physical Therapy, China Medical University, Taichung, Taiwan, R.O.C.; 6 Department of Orthopaedic Surgery, China Medical University, Taichung, Taiwan, R.O.C.

**Keywords:** Statistical shape model-based model, Asynchronous fluoroscopy image, 3D/2D image registration, Knee kinematics

## Abstract

**Background:**

A 2D fluoroscopy/3D model-based registration with statistical shape modeling (SSM)-reconstructed subject-specific bone models will help reduce radiation exposure for 3D kinematic measurements of the knee using clinical alternating bi-plane fluoroscopy systems. The current study aimed to develop such an approach and evaluate in vivo its accuracy and identify the effects of the accuracy of SSM models on the kinematic measurements.

**Methods:**

An alternating interpolation-based model tracking (AIMT) approach with SSM-reconstructed subject-specific bone models was used for measuring 3D knee kinematics from dynamic alternating bi-plane fluoroscopy images. A two-phase optimization scheme was used to reconstruct subject-specific knee models from a CT-based SSM database of 60 knees using one, two, or three pairs of fluoroscopy images. Using the CT-reconstructed model as a benchmark, the performance of the AIMT with SSM-reconstructed models in measuring bone and joint kinematics during dynamic activity was evaluated in terms of mean target registration errors (mmTRE) for registered bone poses and the mean absolute differences (MAD) for each motion component of the joint poses.

**Results:**

The mmTRE of the femur and tibia for one image pair were significantly greater than those for two and three image pairs without significant differences between two and three image pairs. The MAD was 1.16 to 1.22° for rotations and 1.18 to 1.22 mm for translations using one image pair. The corresponding values for two and three image pairs were 0.75 to 0.89° and 0.75 to 0.79 mm; and 0.57 to 0.79° and 0.6 to 0.69 mm, respectively. The MAD values for one image pair were significantly greater than those for two and three image pairs without significant differences between two and three image pairs.

**Conclusions:**

An AIMT approach with SSM-reconstructed models was developed, enabling the registration of interleaved fluoroscopy images and SSM-reconstructed models from more than one asynchronous fluoroscopy image pair. This new approach had sub-millimeter and sub-degree measurement accuracy when using more than one image pair, comparable to the accuracy of CT-based methods. This approach will be helpful for future kinematic measurements of the knee with reduced radiation exposure using 3D fluoroscopy with clinically alternating bi-plane fluoroscopy systems.

## Introduction

Accurate measurement of the 3D kinematics of the knee during activities is indispensable for evaluating the function of the joint and for the diagnosis of injuries or diseases (Lu et al., 2008; Zantop et al., 2008). It is also helpful for assessing the efficacy of clinical interventions (Brandsson et al., 2002) and for designing personalized prostheses or surgical procedures (Eck et al., 2002; Kleipool & Blankevoort, 2010; McEwen et al., 2005; Wilson, Feikes & O’Connor, 1998). X-ray imaging systems have been used in combination with CT model-based 3D/2D image registration techniques for non-invasive measurements of 3D kinematics of normal (Lin et al., 2014a; Lu et al., 2008), pathological (Ikuta et al., 2020; Kobayashi et al., 2015; Lynch et al., 2020) and replaced joints (Dai et al., 2021; Kono et al., 2021; Kono et al., 2020; Shih et al., 2020) during activities. Although these approaches have reasonably high accuracy for 3D kinematic measurement, the concern of the radiation exposure needed for getting CT-based bone models has limited its routine applications in clinical settings. Alternative approaches to obtain subject-specific bone models are needed for more widespread clinical use of 3D/2D registration methods for knee kinematics.

Model-based 3D/2D registration methods for knee kinematics measurements were first proposed using single-plane fluoroscopy (Banks & Hodge, 1996; Tsai et al., 2010). However, the measurement accuracy for translations normal to the image plane is substantially less than those of the other in-plane components (Fregly, Rahman & Banks, 2005; Lin et al., 2014b). Although techniques have been developed to improve the out-of-plane accuracy (Lin et al., 2014a), the state-of-the-art bi-plane flat-panel fluoroscopy has provided higher accuracy than single-plane methods (Lin et al., 2014b). Methods based on biplane fluoroscopy required synchronized imaging by the two fluoroscopy panels, which were achieved only on custom-built biplane systems or by integrating two single-plane C-arm fluoroscopes (Anderst et al., 2009; Brainerd et al., 2010; Campbell et al., 2016; Kapron et al., 2014; Koo, Lee & Cha, 2015). However, in clinical settings such as angiography rooms and operating theatres commercially available interleaved bi-plane fluoroscopy systems, *i.e.,* the two image planes are working alternately, are widely used because the time lag between the two X-ray units diminishes the effects of Compton Scatting as well as radiation exposures. Such interleaved bi-plane imaging systems pose a major issue in their applications in measuring human joint motions. Such temporal asynchronization leads to errors in the 3D/2D registered bone poses in most registration methods developed for synchronized biplane images (Akbari-Shandiz et al., 2018; Lin et al., 2018). To address this limitation, a new model-based tracking method with alternating between-frame interpolation strategies, here referred to as alternating interpolation-based model tracking (AIMT), was proposed (Lin et al., 2020). The AIMT approach was introduced with strategies related to alternating between-frame kinematic interpolations (Akbari-Shandiz et al., 2018; Lin et al., 2020; Lin et al., 2018). The 3D bone poses in neighboring frames of an image plane obtained using single-plane image registration were used to generate a central interpolated bone pose synchronized with the image of the other image plane. The synchronized bone pose and image were used to obtain the final bone pose *via* a two-level image registration procedure, giving sub-millimeter and sub-degree accuracy in the measured bone and joint poses (Lin et al., 2018). These approaches relied on successful single-plane image registrations for alternating image frames. A recently improved AIMT approach was proposed to eliminate the need for single-plane image registrations. For each alternating image frame, a pseudo-synchronous image pair for both image planes was generated by between-frame image interpolations. This approach tackled the problem of interleaved bi-plane fluoroscopy imaging. Also, it enabled the automation of the entire model-based tracking process with a high translation accuracy ranging from 0.11 to 0.35 mm and a high rotation accuracy ranging from 0.18°to 0.49° (Lin et al., 2020).

Another issue with bi-plane fluoroscopy is the radiation exposure in CT scans for establishing subject-specific bone models. MR images may be an alternative approach to obtaining subject-specific models (Lin et al., 2013). However, since MR imaging has less accuracy in the edges of the bone images, the accuracy of the reconstructed bone models is less accurate than CT-based models (Markelj et al., 2012; Moro-oka et al., 2007). Accurate and rapid reconstruction of 3D personalized bone models using statistical shape modeling (SSM) techniques with planar radiographs has excellent potential to address the radiation dosage problem and reduce cost. SSM techniques have been used in the development of fully automated bone segmentation methods (Fripp et al., 2006; Josephson, Ericsson & Karlsson, 2005; Lamecker et al., 2004), parametric descriptions of the bony geometry (Seber, 2009; Zhu & Li, 2011) and semi-automatic reconstruction of subject-specific bone models (Baka et al., 2011). CT-based SSM model of the knee enabled the reconstruction of subject-specific bone models from 2 or more planar fluoroscopy images (Baka et al., 2011; Karade & Ravi, 2015; Lamecker, Wenckebach & Hege, 2006; Sarkalkan, Weinans & Zadpoor, 2014; Zhu & Li, 2011). A recent two-phase optimization approach based on registering pseudo DRRs to multiple views of fluoroscopic images have shown submillimeter accuracy in reconstructing subject-specific 3D shape model for the femur and tibia (Lu et al., 2021). The results showed that two and three image pairs had better 3D shape reconstruction accuracy than one pair. Considering computational costs, two image pairs may be preferred over three image pairs (Lu et al., 2021). It remains unclear whether such SSM-reconstructed models would give accurate bone motions obtained using 3D/2D registration.

Previous studies utilized synchronized biplane fluoroscopy and SSM-based bone models to obtain the 3D knee kinematics of joints. However, only a few studies evaluated their accuracies in knee kinematics measurements. Baka et al. (2014) proposed a surface model-based 3D/2D registration method by optimizing simultaneously the shape and pose of the SSM-based surface bone models to match multiple fluoroscopy images of knee motions during drop landing. With a custom-built synchronized biplane fluoroscopy system, they could use multiple fluoroscopic images for bone shape reconstruction and pose estimation, giving high measurement accuracy compared to bone-marker-based kinematic measurements (Baka et al., 2014). The study suggests that multiple synchronized images help improve the 3D/2D registration accuracy of 3D kinematics of subject-specific bone models. The 3D/2D registration using a synchronous bi-plane fluoroscopy system for 3D kinematics of the SSM-based model was obtained by minimizing the distance between bony contours of the femur and tibia on the fluoroscopic images and the projections of the 3D surface models (Li et al., 2014). The root-mean-square errors (RMSE) between SSM-based and CT-based surface models were 1.16 and 1.4 for the femur and tibia, respectively, and the RMSE in the registered kinematics was 3.3° in rotation and 2.4 mm in translation (Li et al., 2014). However, the performance of these approaches may be compromised when using clinically available alternating asynchronized fluoroscopy images. More recent computer simulation studies used multiple image pairs for model reconstruction, and kinematics measurement (Smoger et al., 2017; Valenti et al., 2016a; Valenti et al., 2016b), but the methods proposed in these studies were limited to the use of synchronous images. To the authors’ best knowledge, no study has reported the use of multiple static views of fluoroscopic images from clinically available alternating bi-plane fluoroscopy systems for 3D kinematics measurements. The influence of the surface accuracy of the SSM-based models due to reconstruction from different image views on the accuracy of model-based 3D/2D image registration of knee kinematics has not been investigated.

The current study aimed to evaluate *in vivo* the accuracy of 3D skeletal kinematics of the knee obtained using an AIMT technique for 3D/2D model-based registration with interleaved bi-plane fluoroscopic images and SSM-reconstructed subject-specific bone models; and to identify the effects of the number of fluoroscopic images used for SSM-reconstructed models, *i.e.,* the accuracy of model shapes, on the kinematic measurements. It was hypothesized that the number of image pairs for SSM model reconstruction would affect the kinematic measurement differences between the SSM model and CT model-based registrations and that increasing image pairs would reduce such differences. It was hoped that the current study would help reduce radiation exposure in kinematic measurements of the knee using 3D fluoroscopy with clinically available alternating bi-plane fluoroscopy systems.

## Materials & Methods

**Figure 1:**
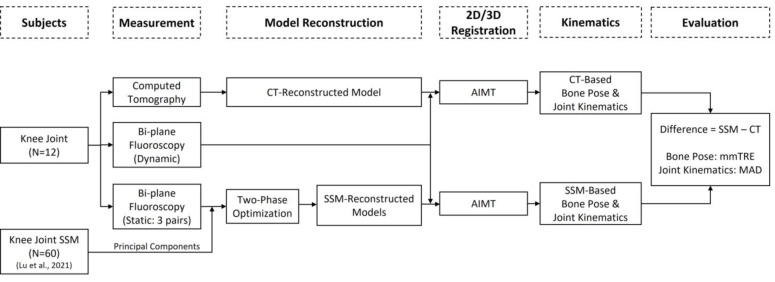
The general procedure for the experimental protocol, SSM of the knee, 3D/2D registration using AIMT with SSM-reconstructed and CT-reconstructed models, and SSM-CT performance difference evaluations in the current study.

The general procedure for the experimental protocol, SSM of the knee, 3D/2D registration using AIMT with SSM-reconstructed and CT-reconstructed models, and SSM-CT performance difference evaluations in the current study are outlined in the flowchart in Fig. 1 and described as follows.

### Subjects

Twelve healthy male volunteers (age: 30.3 ± 17.1 years; height: 172.4 ± 7.0 cm; body mass: 64.5 ± 9.5 kg) without any neuromusculoskeletal disease or surgical history of the lower limbs participated in the current study. The subjects were fully informed of the experimental protocol approved by the Institutional Review Board and gave their written consent (China Medical University Hospital Research Ethics Committee, No: CMUH107-REC2-078). All subjects were scanned by CT at a voxel size of 0.709 mm ×0. 709 mm ×0.625 mm (Optima CT660; GE Healthcare, Chicago, IL, USA) for the reconstruction of subject-specific volumetric models of the knee, which were not involved in building the knee SSM reported by (Lu et al. (2021)).

### Experimental protocol

The subjects performed active flexion and extension of the left knee with the right leg supporting the body weight. In addition to the tested tasks, all subjects performed static standing for subject calibration, from which the knee pose was obtained as the baseline for subsequent analysis. A clinical bi-plane fluoroscopy system (Allura XPER FD 20/20; Philips Medical Systems, Best, Netherlands) was used to acquire the interleaved bi-plane X-ray images of the knee during tested tasks at a resolution of 512 × 512, a grayscale of 8-bit, and an effective frame rate of 60 fps with a constant time interval of 1/60 s between two X-ray units. The two fluoroscopic detector units were positioned orthogonally with each other. Each fluoroscopic unit was modeled as an ideal perspective projection of a point source of X-ray. Before data acquisition, a well-established experimental calibration procedure was performed to obtain the intrinsic and extrinsic parameters for each projection model of the fluoroscopy units (Lin et al., 2014b; Lin et al., 2018). Four lead skin markers were attached to the distal thigh and proximal shank, and one lead marker was attached to the patella to determine the spatial transformation between the X-ray image pairs. For each pair of fluoroscopy images, the 3D coordinates of the lead markers were first determined using radio-stereometric analysis (Karrholm, 1989). The obtained coordinates of the lead markers from any two image pairs were then co-registered to determine the transformations between the two image pairs. Each subject stood with the tested foot on a rotating plate and the left knee located at the isocenter of the bi-plane imaging system during subject calibration. Multiple views of fluoroscopic images were acquired by rotating the tested lower limb vertically around the isocenter of the bi-plane fluoroscope for 0°, 30°, 45°, and 60°. From these positions, three combinations of asynchronous X-ray image pairs were used for the subsequent reconstruction of personalized knee model: (1) one image pair (two orthogonal images from the 0° position); (2) two image pairs (four images from the 0° and 45° positions); (3) three image pairs (six images from the 0°, 30°, and 60° positions).

### Statistical shape modeling of the knee

The general procedure of the knee SSM included (1) obtaining a set of CT-derived training shape models, (2) choosing a reference model with a predefined surface mesh; (3) establishing shape (mesh) correspondence between individual training models by transforming the reference model to individual training ones; and (4) determining the mean model and primary modes of shape variations using Principal Component Analysis (PCA). The training shape models for the SSM were reconstructed from the CT data of the distal femur and the proximal tibia from 60 healthy Chinese males (Lu et al., 2021). The mesh of the femoral reference model had 3524 vertices, and that of the tibial reference model had 1901 vertices. Individual training models were obtained by co-registering the reference and CT-based models *via* shape correspondence using the iterative closest point (ICP) (Besl & McKay, 1992) and coherent point drift (CPD) (Myronenko & Song, 2010) methods, and shape alignment using Generalized Procrustes analysis (GPA) (Cootes & Taylor, 2004). The variations of the individual shape models were then decomposed to a set of principal components (eigenvectors) using principal component analysis (PCA) (Wold, Esbensen & Geladi, 1987). Thus, each training shape model could be described as the mean model superimposed with a linear combination of the principal components.

For each of the current 12 knee joints, the subject-specific bone shape model was generated using the trained SSM with multiple asynchronous 2D images of the bone *via* a two-phase optimization scheme, referred to as SSM-reconstructed model (Lu et al., 2021). In the two-phase optimization scheme, the first phase involved searching for the optimum pose (*i.e.,* six degrees of freedom of the bone) and shape (*i.e.,* parameters for the first 10 principal components) that maximizes the similarity between the pseudo digitally reconstructed radiographs (DRRs) of the SSM-reconstructed bone model and the multiple asynchronous 2D fluoroscopy images (Lu et al., 2021). Generally, a DRR was generated by casting rays from the X-ray point source towards the image plane through the CT-reconstructed model, from which the interior voxel values (*i.e.,* the ideal attenuation coefficients) of the bone encountered by each of these rays were accumulated to derive the grayscale values of the DRR (Engel et al., 2006). For generating the DRRs of an SSM-reconstructed bone model, the model was transformed to a pseudo volumetric one by a voxelization process, which was achieved by first finding intersections between the shape model and a set of parallel virtual 2D transverse slices (pixel size of 0.5 mm ×0.5 mm and inter-slice distance of 0.5 mm) (Patil & Ravi, 2005). The resulting virtual voxels interior to the shape model were assigned a constant of 700 to simulate the Hounsfield unit (HU) value of bone, while voxels outside the contours were assigned −1.000 to simulate air. As a result, the DRRs of the pseudo volumetric SSM-reconstructed models in space were generated for a given bone pose and shape. In the second phase, the shape model and its pose obtained in the first phase were further refined. The shape model was further refined by including ten additional shape parameters (*i.e.,* 11th to 20th principal components) to better match the fluoroscopic images taking the pose and shape parameters obtained in the first phase as fixed parameters, giving the final shape of the SSM-reconstructed bone model (Lu et al., 2021). The genetic algorithm (Goldberg & Holland, 1988) was employed to search for the optimal poses and shape coefficients parameters. By partitioning the shape and pose parameters, the two-phase optimization approach enabled the consideration of more principal components with increased accuracy but reduced computational effort compared to a single-phase optimization with 10 principal components (Lu et al., 2021).

### 3D/2D registration using AIMT with SSM-reconstructed and CT-based models

**Figure 2:**
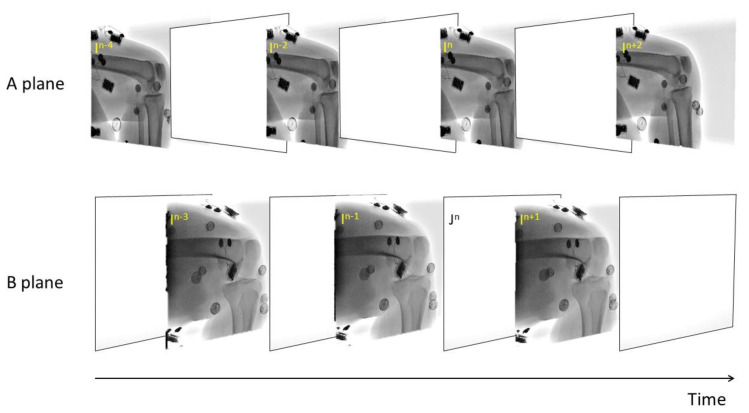
A series of interleaved biplane fluoroscopic images generated from two X-ray units of an alternating bi-plane fluoroscopy system.

**Figure 3:**
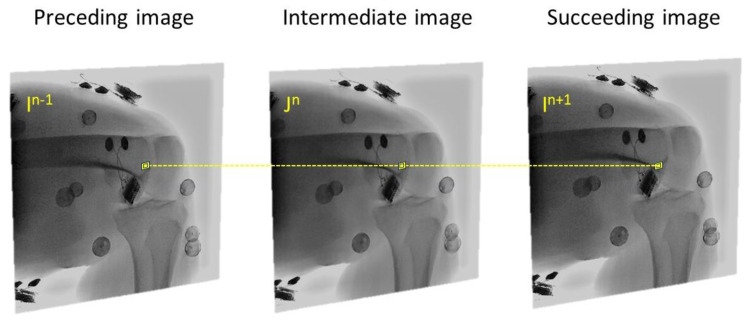
The intermediate image (*J*^*n*^) at time point n of B X-ray unit could be reconstructed from the preceding (I^*n*−1^) and succeeding image (I^*n*+1^) using the motion-compensated frame interpolation method.

**Figure 4:**
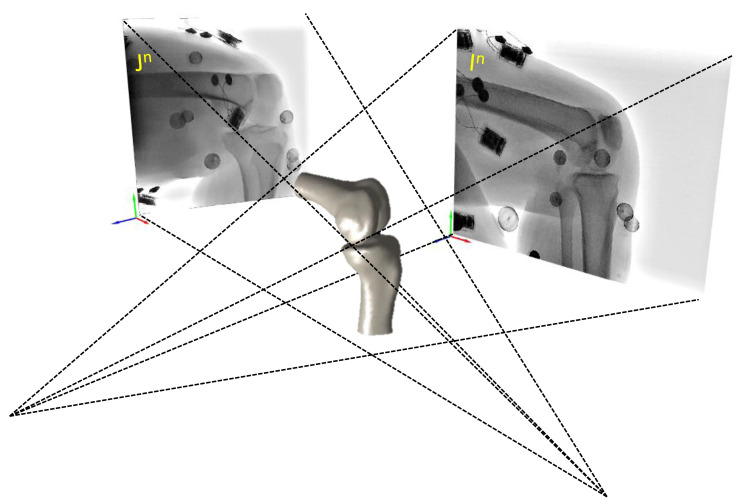
The interpolated image I^*n*^ and J^*n*^ of A X-ray unit constitute a synchronous biplane fluoroscopy imaging system used to obtain the six-degree-of-freedom bone poses of the SSM-reconstructed and CT-based subject-specific bone models.

We utilized a validated AIMT approach (Lin et al., 2020) to accomplish the fully automated kinematics tracking of the knee, from which the SSM-reconstructed and CT-reconstructed subject-specific bone models were separately registered to the interleaved bi-plane fluoroscopic images to obtain the six-degree-of-freedom pose parameters (Fig. 2). The AIMT technique consists of three stages, namely (1) 2D/2D template registration, (2) motion-compensated frame interpolation, and (3) bi-plane 3D/2D image registration. The first stage analysis aimed to estimate the bone model’s pose parameters for the current frame from the registered pose in the preceding frame. This was accomplished by estimating the 2D pose increment between the preceding and current image frames using template registration with the particle filter (Arulampalam et al., 2002). The 2D pose increments from the two fluoroscopic views were then summed up to obtain the 3D pose increment used to estimate the pose parameters of the bone model in the current frame (Lin et al., 2020). In the second stage, the interleaved bi-plane images were converted into pseudo synchronous bi-plane image pairs. To this end, an intermediate image frame (I^n^) between any two consecutive image frames I ^n−1^ and I ^n+1^ in the same fluoroscopic view was synthesized using a motion-compensated frame interpolation method (Zhai et al., 2005) (Figs. 2 and 3. In the final stage, a bi-plane model-based 3D/2D image registration scheme based on the forward projection model was implemented to precisely determine the pose parameters of the bones (Fig. 4). The 3D pose parameters of the bone were determined by maximizing the normalized cross-correlation between gradients (*i.e.,* so-called gradient correlation) (Penney et al., 1998). Optimization procedure for 3D/2D image registration was performed to minimize a metric of gradient correlation (GC, *f*_*GC*_) that quantified the similarity of pixel intensity between the model-projection DRRs (*I*_*DRR*_) and the pseudo-bi-plane fluoroscopic images (*I*_*Fluoro*_). DRR image (*I*_*DRR*_) and fluoroscopic image (*I*_*Fluoro*_) are transformed by applying horizontal (*i*) and vertical Sobel templates (*j*) to obtain four gradient images, namely *dI*_*DRR*_/*di*, *dI*_*DRR*_/*dj*, *dI*_*fl*_/*di*, *dI*_*fl*_/*dj*. GC is defined with normalized cross correlation between *I*_*Fluoro*_ and *I*_*DRR*_ as the follows Eq. (1). The sum of −*f*_*GC*_ was taken as the cost function to be minimized by using N number of alternating images (*N* = 2) with design variables of six degrees of freedom (*x*, *y*, *z*, *α*, *β*, *γ*) Eq. (2). The genetic algorithm was employed to search for the optimal poses (Goldberg & Holland, 1988). Noted that the pose parameters reproduced using the CT-derived bone models were taken as the standard reference for evaluating the proposed method.

\begin{eqnarray*}{f}_{GC}& & = \frac{\sum _{i}[d{I}_{fl}/di-{\overline{d{I}_{fl}/di}}_{Fluoro}]\sum _{i}[d{I}_{DRR}/di-{\overline{d{I}_{DRR}/di}}_{DRR}]}{\sqrt{\sum _{i}[d{I}_{fl}/di-{\overline{d{I}_{fl}/di}}_{Fluoro}]^{2}}\sqrt{\sum _{i}[d{I}_{DRR}/di-{\overline{d{I}_{DRR}/di}}_{DRR}]^{2}}} \end{eqnarray*}

(1) \begin{eqnarray*}& & + \frac{\sum _{j}[d{I}_{fl}/dj-{\overline{d{I}_{fl}/dj}}_{Fluoro}]\sum _{j}[d{I}_{DRR}/dj-{\overline{d{I}_{DRR}/dj}}_{DRR}]}{\sqrt{\sum _{j}[d{I}_{fl}/dj-{\overline{d{I}_{fl}/dj}}_{Fluoro}]^{2}}\sqrt{\sum _{j}[d{I}_{DRR}/dj-{\overline{d{I}_{DRR}/dj}}_{DRR}]^{2}}} \end{eqnarray*}

where ${\overline{d{I}_{fl}/di}}_{Fluoro}$, ${\overline{d{I}_{DRR}/di}}_{DRR}$, ${\overline{d{I}_{fl}/dj}}_{Fluoro}$, ${\overline{d{I}_{DRR}/dj}}_{DRR}$ are the mean values of the transformed images.

(2) \begin{eqnarray*}{f}_{\mathit{GC},\mathit{all}}=\arg \nolimits \min _{{f}_{\mathit{GC}}}\sum _{p=1}^{N}{ \left( x,y,z,\alpha ,\beta ,\gamma \right) }_{p}\end{eqnarray*}

From the registered poses of the bones, the knee kinematics were then calculated. The anatomical coordinate systems (ACS) of the CT-based models of the femur and tibia were determined by the 3D geometry features of the bone models (Miranda et al., 2010), with the positive *x*-axis directed anteriorly, positive *y*-axis directed superiorly and positive *z*-axis directed to the right. The CT-based model was co-registered with the corresponding SSM-reconstructed bone model *via* the ICP method, and the ACS of the CT-based model was then assigned to the registered SSM-reconstructed model. This approach minimized the possible discrepancies in the coordinate systems between SSM- and CT-based models. The knee kinematics was described as the tibial pose in the femoral ACS. The joint angles were computed using a *z* − *x* − *y* Cardanic sequence (Grood & Suntay, 1983), giving flexion/extension, abduction/adduction and external/internal rotations.

### Evaluation of the AIMT technique with SSM-reconstructed bone model

For the quantification of the shape differences between the SSM-reconstructed model and the corresponding CT-reconstructed model, the models were first spatially aligned to each other *via* the ICP method (Besl & McKay, 1992), and the point-to-surface distance 𝔢 for each point *p* on the surface of the SSM-reconstructed model was then calculated as its shortest Euclidean distance to a corresponding point *p*′ on the surface S of the CT-reconstructed bone model as follows (Cignoni, Rocchini & Scopigno, 1998).

(3) \begin{eqnarray*}e \left( \mathrm{p},\mathrm{S} \right) = \left\vert \min _{p{^{\prime}}\epsilon S}~d(p,p{^{\prime}}) \right\vert \end{eqnarray*}

The root-mean-squared values of 𝔢 over the entire surface points (RMSe) were obtained as a measure of the shape error of the SSM-reconstructed model.

For the quantification of the differences between the registered bone kinematics during the tested dynamic activity using SSM-reconstructed and CT-reconstructed models, the mean target registration error (mTRE) (Van de Kraats et al., 2005) was calculated as the mean of 𝔢 values over the entire SSM-reconstructed model surface for each image frame. The mean of mTRE (mmTRE) was then obtained by averaging the mTRE of each frame over the dynamic motion cycle. The peak mTRE (pmTRE) over the dynamic motion cycle was also obtained. In this study, a successful registration was defined as mTRE less than 1.5 mm.

The differences between the registered joint poses using the SSM-reconstructed and CT-reconstructed models were quantified by the mean absolute differences (MAD) in each of the six kinematic components (*i.e.,* three translations and three rotations) over the dynamic motion cycle. The peak absolute differences (PAD) over the dynamic motion cycle was also obtained for each kinematic component. The mean and standard deviation (SD) of the MAD across all the subjects gave the bias and precision of the model used, respectively.

Computational costs (time) in reconstructing SSM-based models from 1, 2 and 3 static image pairs and registering these SSM-based models to interleaved biplane fluoroscopic images over the tested dynamic activity were also obtained on a 3.7 GHz, 32 GB computer running a MATLAB implementation.

### Statistical analysis

One-way ANOVA was used to test the differences in mmTRE, MAD in six-degree-of-freedom components, computational costs in reconstructing SSM-based models from 1, 2 and 3 image pairs and registering SSM-based models to fluoroscopic images with paired *t*-tests by using Bonferroni correction for *post hoc* pairwise comparisons. All comparisons were performed using SPSS 20.0 (SPSS, IBM, Armonk, New York, NY, USA). The significance level was set at *α* = 0.05.

## Results

The mean (SD) of the RMSe for the shape errors of the SSM-reconstructed model using one image pair over all subjects for the femur and tibia were 0.83 (0.13) mm and 0.84 (0.11) mm, respectively. The corresponding values for two and three image pairs were 0.7 (0.09) mm and 0.71 (0.05) mm, and 0.66 (0.09) mm and 0.67 (0.07) mm, respectively (Table 1). The shape errors (RMSe) for the femur and tibia using one image pair were significantly greater than those using two or three image pairs, but no significant differences were found between two and three image pairs. Also, computational efficiency in reconstructing SSM-based model of femur using one, two and three image pairs were 73.39 (7.42) s, 76.72 (7.31) and 86.89 (7.27) s, respectively. The corresponding values for tibia were 57.85 (3.34), 70.09 (4.63) and 79.15 (5.39) s (Table 1). No significant differences in computational efficiency were found between different image pairs for reconstructing SSM-based model of femur and tibia.

**Table 1:** Means (standard deviations) of the root-mean-squared values of 𝔢 (RMSe) and computational efficiency in reconstructing SSM-based models of the femur and tibia from a different number of image pairs using the two-phase optimization method.

		1 image pair	2 image pairs	3 image pairs	*P*_*A*_	*P*_*B*_	*P*_*C*_
Femur	RMSe (mm)	0.83 (0.13)	0.70 (0.09)	0.66 (0.09)	0.025^∗^	0.003^∗^	0.64
	Time (s)	73.39 (7.42)	76.72 (7.31)	86.89 (7.27)	0.512	<0.001^∗^	0.005^∗^
Tibia	RMSe (mm)	0.84 (0.11)	0.71 (0.05)	0.67 (0.07)	0.005^∗^	<0.001^∗^	0.559
	Time (s)	57.86 (3.34)	70.09 (4.63)	79.15 (5.39)	<0.001^∗^	<0.001^∗^	<0.001^∗^

**Notes.**

*P* values: *P*_*A*_ = 1 vs. 2 image pairs; *P*_*B*_ = 1 vs. 3 image pairs; *P*_*C*_ = 2 vs. 3 image pairs.

**Figure 5:**
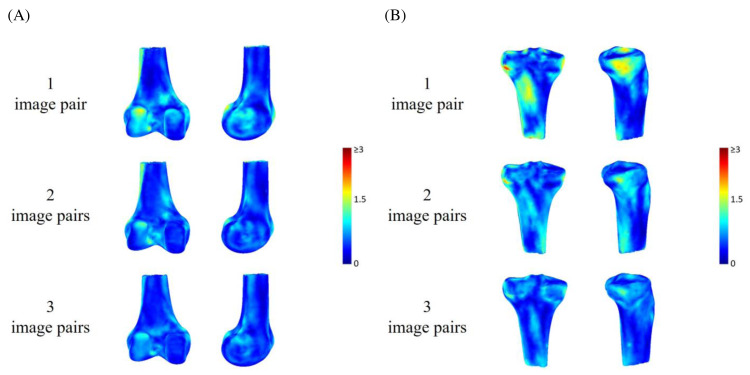
Pose errors of the (A) femur and (B) tibia using rendering to indicate the mean of mean target registration error (mmTRE) of the SSM-based model by using one, two and three image pairs.

**Figure 6:**
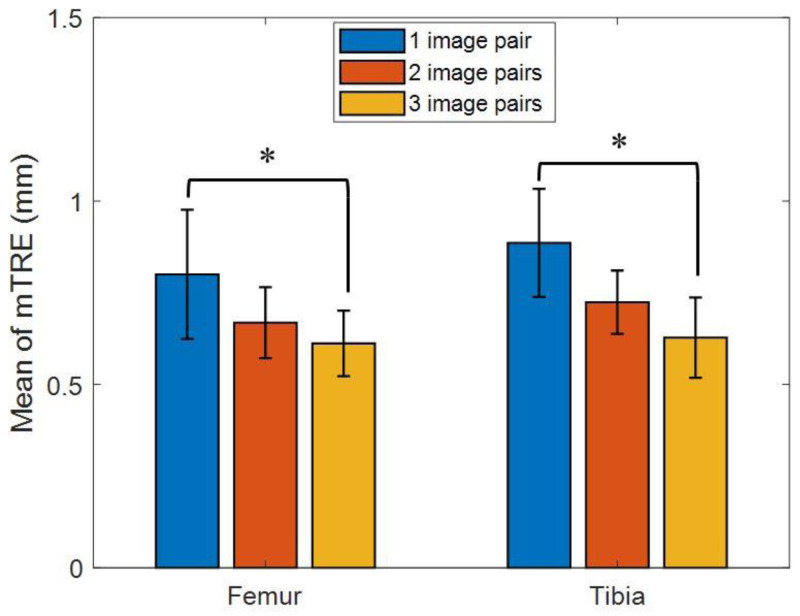
Mean of mean target registration error (mmTRE) for femur and tibia between CT-based and SSM-based bone model by using one (blue bar), two (red bar), and three image pairs (yellow bar) during active knee flexion and extension. An asterisk (*) indicates a significant difference with *α* = 0.05.

The means (SD) of mmTRE of the registered poses of the femur and tibia using one image pair across all the subjects were 0.8 (0.18) mm and 0.89 (0.15) mm during active knee flexion and extension. The corresponding values for two and three image pairs were 0.67 (0.10) mm and 0.72 (0.09) mm, and 0.61 (0.09) mm and 0.63 (0.11) mm, respectively (Table 2 and Fig. 5). The mmTRE of the registered poses of the femur and tibia using one image pair were significantly greater than those using two or three image pairs, but no significant differences were found between two and three image pairs (Fig. 6). The means (SD) of pmTRE for the femoral and tibial poses using one image pair were 0.87 mm (0.23) and 1.06 mm (0.19) across all the subjects. The corresponding values for two and three image pairs were 0.73 mm (0.17) and 0.92 mm (0.2); and 0.6 mm (0.12) and 0.69 mm (0.13), respectively. Computational costs in registering SSM-based model of the femur, reconstructed from 1, 2 and 3 image pairs, to bi-plane fluoroscopic images for an image frame were 22.21 (5.46) s, 22.03 (5.87) s and 25.84 (4.91) s, respectively. The corresponding values for tibia were 18.64 (4.2) s, 20.59 (6.32) s and 23.08 (4.0) s (Table 2). No significant differences in registration efficiency were found between SSM-based models using different image pairs of femur and tibia.

**Table 2:** The mean and standard deviation of pose errors and registration efficiency of the SSM-based bone model between one, two and three image pairs during active knee flexion and extension.

		1 image pair	2 image pairs	3 image pairs	*P*_*A*_	*P*_*B*_	*P*_*C*_
Femur	mmTRE (mm)	0.80 (0.18)	0.67 (0.10)	0.61 (0.09)	0.016^∗^	<0.001^∗^	0.282
	Time (s)	22.21 (5.46)	22.03 (5.87)	25.84 (4.91)	0.996	0.217	0.188
Tibia	mmTRE (mm)	0.89 (0.15)	0.72 (0.09)	0.63 (0.11)	0.002^∗^	<0.001^∗^	0.051
	Time (s)	18.64 (4.2)	20.59 (6.32)	23.08 (4.0)	0.557	0.059	0.387

**Notes.**

*P* values: *P*_*A*_ = 1 vs. 2 image pairs; *P*_*B*_ = 1 vs. 3 image pairs; *P*_*C*_ = 2 vs. 3 image pairs.

**Figure 7:**
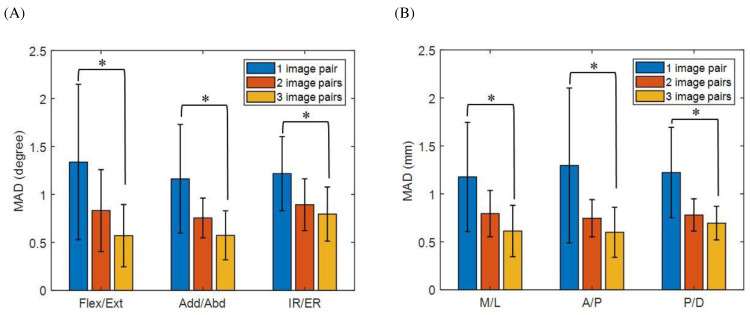
Mean absolute error (MAD) of knee joint kinematics in flexion/extension, adduction/abduction, internal/external rotation angles (A) and medial/lateral, anterior/posterior, proximal/distal translations (B) by using SSM-based model between one (blue bar), two (red bar) and three image pairs (yellow bar). An asterisk (*) indicates a significant difference with *α* = 0.05.

**Figure 8:**
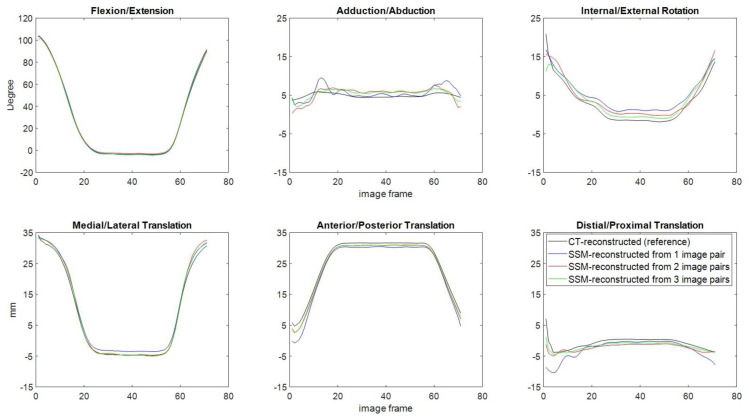
Knee kinematics in six components (flexion/extension, abduction/adduction and internal/external rotations, and medial/lateral, anterior/posterior and distal/proximal translations) of a typical subject obtained using the AIMT approach with SSM models reconstructed using one (blue), two (red) and three image pairs (green), and with CT-reconstructed model (black) during flexion/extension.

For the joint pose differences between the CT-reconstructed model and the SSM-reconstructed knee using one image pair, the means (SD) of MAD in flexion/extension, abduction/adduction, internal/external rotations, and medial/lateral, anterior/posterior, and proximal/distal translations were 1.34 (0.81°), 1.16 (0.57°), 1.22 (0.39°), 1.18 (0.57) mm, 1.29 (0.81) mm and 1.22 (0.47) mm, respectively (Fig. 7 and Table 3). The corresponding values for two and three image pairs were 0.83 (0.43°) and 0.57 (0.32°), 0.75 (0.21°) and 0.57 (0.26°), 0.89 (0.27°) and 0.79 (0.28°), 0.79 (0.24) mm and 0.61 (0.27) mm, 0.75 (0.19) mm and 0.6 (0.26) mm, 0.78 (0.17) mm and 0.69 (0.18) mm (Fig. 7 and Table 3). The means (SD) of PAD in flexion/extension, abduction/adduction, internal/external rotations, and medial/lateral, anterior/posterior, and proximal/distal translations for one image pair across all the subjects were 3.58° (1.26), 3.57° (1.29), 4.19° (1.66), 3.26 mm (0.73), 3.68 mm (1.18) and 4.1 mm (1.32), respectively. The corresponding values for 2 and 3 image pairs were 1.87° (1.63) and 1.34° (0.42), 1.84° (1.08) and 1.59° (0.69), 2.08° (1.12) and 1.96° (0.93), 1.56 (0.37) mm and 1.46 (0.45) mm, 1.94 mm (0.39) and 1.2 (0.22) mm, 1.54 mm (0.44) and 1.44 mm (0.35). The effect power of the current results was 0.87 with a large effect size (partial *η*2 = 0.261), evaluated by a *post hoc* power analysis using G*POWER (Erdfelder, Faul & Buchner, 1996). The mean MAD in rotations and translations of the SSM-reconstructed knee using one image pair (rotations: 1.24°; translations: 1.23 mm) were significantly greater than those using two image pairs (rotations: 0.83°; translations: 0.77 mm) and three image pairs (rotations: 0.65°; translations: 0.64 mm), while no significant differences were found between two and three image pairs (Fig. 7). The time histories of the knee kinematic components of a typical subject obtained using SSM-reconstructed models and CT-reconstructed models during the flexion-extension activity is shown in Fig. 8.

**Table 3:** Means (standard deviations) of the mean absolute differences (MAD) of six-degree-of-freedom in bias and precision between the gold standard of CT-based and reconstructed SSM-based using one, two and three image pairs during knee flexion and extension.

Six degree-of-freedom	1 image pair	2 image pairs	3 image pairs	*P*_*A*_	*P*_*B*_	*P*_*C*_
Flexion/Extension (°)	1.34 (0.81)	0.83 (0.43)	0.57 (0.32)	0.034^∗^	0.002^∗^	0.263
Adduction/Abduction (°)	1.16 (0.57)	0.75 (0.21)	0.57 (0.26)	0.012^∗^	<0.001^∗^	0.25
Internal/External rotation (°)	1.22 (0.39)	0.89 (0.27)	0.79 (0.28)	0.018^∗^	0.003	0.457
Medial/Lateral translation (mm)	1.18 (0.57)	0.79 (0.24)	0.61 (0.27)	0.022^∗^	0.001^∗^	0.261
Anterior/Posterior translation (mm)	1.29 (0.81)	0.75 (0.19)	0.60 (0.26)	0.011^∗^	0.002^∗^	0.48
Proximal/Distal translation (mm)	1.22 (0.47)	0.78 (0.17)	0.69 (0.18)	0.001^∗^	<0.001^∗^	0.502

**Notes.**

*P* values: *P*_*A*_ = 1 vs. 2 image pairs; *P*_*B*_ = 1 vs. 3 image pairs; *P*_*C*_ = 2 vs. 3 image pairs.

## Discussion

The current study aimed to evaluate *in vivo* the accuracy of 3D skeletal kinematics of the knee obtained using a validated AIMT technique for 2D interleaved fluoroscopy/3D model-based image registration (Lin et al., 2020) with SSM-reconstructed bone models from asynchronous fluoroscopy images (Lu et al., 2021) and to quantify the effects of the number of the asynchronous fluoroscopic image pairs on the kinematic measurement accuracy. Measurements using CT-based models were used as a benchmark for assessment. The registration method using SSM-based models reconstructed from a single pair of fluoroscopy images gave mean differences of slightly more than one mm in translations and one degree in rotations from the benchmark. Registrations using SSM-based models reconstructed from two or three image pairs achieved much better accuracy than a single image pair with sub-millimeter and sub-degree accuracy. The accuracy of the 3D subject-specific bone model plays an essential role in the 3D kinematics measurement using the AIMT technique. The current results suggest that SSM-reconstructed models from more than one image pair were accurate enough to produce measurement accuracies comparable to those using CT-based models. Considering both reconstruction quality and computing efficiency, two image pairs would be a better choice as no significant differences were found between two and three image pairs. This will help reduce the radiation exposures of the patients in getting 3D fluoroscopy measurements using clinical settings.

In model-based 3D/2D image registration, the types of bone models (*i.e.,* radiodensity, flat-shading, and homogeneous-density) and the number of fluoroscopic views all contribute to the measurement accuracy of registered bone poses. The CT-based model preserves the radiodensity of the bone, allowing the further generation of DRRs (Siddon, 1985), so it was used to provide the ground truth. When the CT data are not available, the bone silhouette generated from surface models with flat-shading is used (Giphart et al., 2012; Moewis et al., 2012; Smoger et al., 2017; Stentz-Olesen et al., 2017), but the lack of interior information and non-attenuated bone edges of flat-shaded images affect the accuracy of the 3D/2D image registration (Fregly, Rahman & Banks, 2005; Lin et al., 2014b). To overcome this limitation, treating voxel intensities interior to the bony surfaces as homogeneous density has been shown to be a viable alternative to generating synthetic images mimicking CT-generated DRR. This approach can generate measurement accuracy close to those obtained using CT-based radiodensity models (Lin, Lu & Lu, 2021). Therefore, the accuracy of the 3D/2D registration using 3D SSM-reconstructed shape models with homogeneous density will depend on the accuracy of the surface model and the registration method used.

The current study used a newly developed and validated two-phase optimization approach to reconstruct SSM-based subject-specific surface models from fluoroscopy imaging taken at different time instances with different views of multiple fluoroscopic image pairs. This approach used homogeneous density in the SSM-based model reconstruction process, the optimal SSM-based model could be obtained as that gave DRRs that best matched the fluoroscopic image pairs. With this new approach, the errors in SSM-reconstructed models decreased as the number of fluoroscopic image pairs increased (Table 1). Using only one synchronized image pair, the two-phase optimization approach gave a much higher accuracy in the reconstructed subject-specific models (Table 1) than most previously reported methods, which had RMSE ranging from 1.33 mm to 1.68 mm for the femur (Baka et al., 2011; Karade & Ravi, 2015; Laporte et al., 2003). If a single asynchronous fluoroscopy image pair was used for SSM-based model reconstruction, the RMSE was 1.16 mm and 1.4 mm for the femur and tibia, respectively (Li et al., 2014). The two-phase optimization approach enabled SSM-based model reconstruction using more than one asynchronous image pair, which was advantageous over the existing method. The current results showed that sub-millimeter and sub-degree accuracy in six-degree-of-freedom bone and joint poses could be achieved using a two-phase optimization approach with more than one image pair (Fig. 7 and Table 3). The SSM-reconstructed models produced by the two-phase optimization approach were also ready for use with DRR-based 3D/2D registration methods, the AIMT technique in the current study and the effects of image pairs used for the SSM-based model reconstruction on the registered bone and joint poses could be evaluated.

Parametric analysis of SSM was used to reconstruct the 3D surface SSM-based model with the first twenty principal components. The 3D volumetric SSM-based model was then generated by a voxelization process with giving parameters of pixel size, inter-slice distance and constant HU value of cortical bone. The pose of SSM-reconstructed model was obtained by optimizing the design variables of six degree of freedom using 3D/2D image registration with AIMT approach. In this study, 3D kinematics of bone model was measured by using less than thirty parameters in 3D/2D registration with SSM method using clinical interleaved bi-plane fluoroscopy. An efficient method in measuring 3D knee kinematics with SSM-based model from multiple views of fluoroscopic images was developed in this study. Baka et al. (2011) used canny-edge detection method to reconstruct the SSM-based model from a series of fluoroscopic image during jump-landing, and the computational time of this approach was about five min. Zhu & Li (2011) reported the calculation time in reconstructing the femur model using two, four and six image were 101 s, 189 s and 399 s, and the computational time from Tsai et al. (2015) using dual fluoroscopic images to construct the distal femur and tibia were 106.6 s and 79.2 s. Few previous studies showed the computational efficiency in 3D/2D image registration using the SSM-based model. Baka et al. reported that computational time in calculating SSM-based knee kinematics was 2 h during approximately 60 frames. On the other hand, it took 2 min to process 3D/2D registration with a set of bi-plane fluoroscopic images (Baka et al., 2014). The current study showed promising efficiency in reconstructing the 3D SSM-based model for the femur and tibia using a two-phase optimization approach with less than 90 s and 80 s, respectively, and registering the SSM-based knee model to 2D interleaved fluoroscopic images for one image frame with less than 30 s.

The current study assessed the measurement accuracy of an AMIT method for 3D/2D image registration using SSM-reconstructed models from multiple asynchronous fluoroscopic images, two or three image pairs giving better accuracy in model reconstruction than using a single image pair. The effects of errors in model shapes on the registered bone and joint poses could be evaluated with different image pairs for SSM mode reconstruction. Using two image pairs (Lu et al., 2021), the validated AIMT technique for 2D interleaved fluoroscopy/3D model-based image registration (Lin et al., 2020) was found to have better accuracy in joint poses (MAD <0.9° in rotations and <0.8 mm in translations; Fig. 7 and Table 3) than most previous surface model-based registration methods using synchronized biplane fluoroscopic images, which gave median differences ranging from 0.99° to 2° in rotations, and from 0.81 mm to 1.5 mm in translations (Baka et al., 2014; Valenti et al., 2016a). Note that experimental conditions may also affect the 3D/2D image registration errors. For example, the studies by Baka et al. (2014) and Valenti et al. (2016a) used nonorthogonal biplane fluoroscopies, which might affect the errors in the depth direction. With a relatively accurate SSM-reconstructed model (RMSE: 1.16 mm and 1.4 mm for femur and tibia), Li et al. (2014) reported measurement errors with an RMSE of 3.3° in rotations and 2.4 mm in translations using a contour-based registration method with images from an alternating bi-plane fluoroscopy. In contrast, considering the asynchronous nature of the images from alternating bi-plane fluoroscopy, the AIMT approach produced sub-millimeter and sub-degree accuracy in the registered knee kinematic measurements (Lin et al., 2020). The use of DRR-based registration in the AIMT approach also contributed to such high accuracy. DRR-based registration using volumetric models has been shown to give better accuracy in 3D kinematics measurement than contour-based registration using surface models (Lin et al., 2014b). With the AIMT approach for the registration of interleaved fluoroscopy images with SSM-based homogeneous-density models, the registered bone and joint poses were found to have sub-millimeter and sub-degree accuracy when using more than one image pair (Figs. 6–7, Tables 2–3). Such results are very likely a result of the benefits of the AIMT approach and DRR-based registration. The current results suggest that SSM-reconstructed subject-specific models with homogeneous density from multiple asynchronous fluoroscopic images can be used with the AIMT approach to measuring 3D knee kinematics with high accuracy. This approach will be helpful for future kinematic measurements of the knee using 3D fluoroscopy with reduced radiation exposure using widely available clinical bi-plane fluoroscopy systems.

The current study proposed a new approach integrating the AIMT technique and SSM-based subject-specific knee modeling for 3D knee joint kinematic measurement using multiple asynchronous fluoroscopic images. This new approach produced tibiofemoral kinematics in young, healthy male adults comparable to those obtained from CT-based models. These results may be further confirmed *via in vitro* experiments on bone and joint kinematics using an independent measurement system, such as marker-based stereophotogrammetry used in the literature (Lin et al., 2020; Lin et al., 2018). The current study evaluated the performance of up to three image pairs, further study may include more image pairs to test whether an optimal number of more than three image pairs existed when considering both quality and computing efficiency. Further studies will evaluate the performance of the current approach in other populations, including females, older people, or those with diseases or deformities in the knee. The current approach used SSM-based homogeneous-density models to achieve sub-millimeter and sub-degree accuracy in the registered bone and joint poses. Further development of SSM-based radiodensity models may help improve further the accuracy of the model reconstruction and fluoroscopy/model registrations.

## Conclusions

The current study proposed a new approach integrating the AIMT technique and SSM-reconstructed subject-specific knee models for 3D joint kinematic measurements using clinically available interleaved bi-plane fluoroscopy. Using the CT-reconstructed model as a benchmark, this new approach produced registered bone and joint poses with sub-millimeter and sub-degree differences when using SSM models reconstructed from more than one image pair, suggesting an accuracy comparable to the benchmark. The current results suggest that the proposed approach will be helpful for future 3D kinematic measurements of the knee with reduced radiation exposure using interleaved bi-plane fluoroscopy.

##  Supplemental Information

10.7717/peerj.15371/supp-1 Supplemental Information 1 Registered bone poses of knee joint Registered poses of CT-based model and SSM-based model from 1, 2 and 3 image pairs. The raw data shows all fluoroscopic images during static standing, and knee flexion-extension. These data were used for reconstructing SSM-based model and measuring the knee kinematics by using 3D/2D image registration.

## Additional Information and Declarations

### Data Availability

The following information was supplied regarding data availability: The raw measurements are available as a Supplementary File.
